# Reduction of Protein Translation and Activation of Autophagy Protect against PINK1 Pathogenesis in *Drosophila melanogaster*

**DOI:** 10.1371/journal.pgen.1001237

**Published:** 2010-12-09

**Authors:** Song Liu, Bingwei Lu

**Affiliations:** Department of Pathology, Stanford University School of Medicine, Stanford, California, United States of America; University of Minnesota, United States of America

## Abstract

Mutations in PINK1 and Parkin cause familial, early onset Parkinson's disease. In *Drosophila melanogaster*, *PINK1* and *Parkin* mutants show similar phenotypes, such as swollen and dysfunctional mitochondria, muscle degeneration, energy depletion, and dopaminergic (DA) neuron loss. We previously showed that PINK1 and Parkin genetically interact with the mitochondrial fusion/fission pathway, and PINK1 and Parkin were recently proposed to form a mitochondrial quality control system that involves mitophagy. However, the *in vivo* relationships among PINK1/Parkin function, mitochondrial fission/fusion, and autophagy remain unclear; and other cellular events critical for PINK1 pathogenesis remain to be identified. Here we show that PINK1 genetically interacted with the protein translation pathway. Enhanced translation through S6K activation significantly exacerbated *PINK1* mutant phenotypes, whereas reduction of translation showed suppression. Induction of autophagy by Atg1 overexpression also rescued *PINK1* mutant phenotypes, even in the presence of activated S6K. Downregulation of translation and activation of autophagy were already manifested in *PINK1* mutant, suggesting that they represent compensatory cellular responses to mitochondrial dysfunction caused by PINK1 inactivation, presumably serving to conserve energy. Interestingly, the enhanced *PINK1* mutant phenotype in the presence of activated S6K could be fully rescued by Parkin, apparently in an autophagy-independent manner. Our results reveal complex cellular responses to PINK1 inactivation and suggest novel therapeutic strategies through manipulation of the compensatory responses.

## Author Summary

Parkinson's disease is the most common neurodegenerative disease affecting the aging population. Clinically it manifests as tremor, muscle rigidity, slow movement, and postural instability. Parkinson's disease is a chronic disorder, and its occurrence and progression are determined by genetic backgrounds and environmental factors. Although the most common forms of Parkinson's disease, the so-called “idiopathic” forms, generally affect people older than 50, some familial forms of the disease occur before age 40. Mutations in *PINK1* and *Parkin* genes have been associated with the latter forms of Parkinson's disease. The inactivation of PINK1 or Parkin causes dysfunction of mitochondria, the powerhouse of the cell, leading to the degeneration of tissues such as the brain and muscle that have high energy demand. In an effort to understand how genetic mutations in PINK1 result in disease and to find effective ways to intervene, we have performed genetic studies in the model organism *Drosophila melanogaster* and found that reduced protein translation or increased autophagy can efficiently mitigate the phenotypes caused by PINK1 inactivation. Our result suggests that pharmacological manipulations of these newly identified PINK1-interacting pathways may prove beneficial for the treatment of Parkinson's disease.

## Introduction

Parkinson's disease (PD) is the most common neurodegenerative disease affecting movement and currently there is no cure. A pathological hallmark of PD is the reduction of dopamine content in the brain, caused by the selective dysfunction and degeneration of DA neurons in the substantia nigra. The causes of DA neuron loss are complex and likely involve both environmental insults and genetic predisposition. Increasing evidences suggest that mitochondrial dysfunction may be linked to the pathogenesis of both sporadic and familial forms of PD.

Recent genetic studies of rare familial forms of PD identified multiple disease genes, including *PINK1* and *Parkin* [1], [2]. *PINK1* encodes a Ser/Thr kinase with a mitochondrial targeting sequence and is partially localized to the mitochondria [3], [4]. Parkin is an E3 ubiquitin ligase that is largely cytosolic under normal conditions. The inactivation of *Drosophila* orthologs of PINK1 or Parkin resulted in similar phenotypes, with the formation of enlarged, swollen mitochondria preceding muscle degeneration, DA neuron loss and spermatogenesis failure [5]–[7]. Further analysis showed that overexpression (OE) of Parkin could rescue *PINK1* mutant phenotype, but not *vice versa*, suggesting that PINK1 and Parkin may function in the same pathway, with Parkin acting downstream of PINK1 [5]–[7]. Interestingly, promoting mitochondrial fission by either overexpression of mitochondrial fission protein Drp1 or downregulation of mitochondrial fusion proteins Marf (the *D. melanogaster* homolog of mammalian mitofusin) or Opa1 could completely rescue *PINK1* or *Parkin* mutant phenotypes, suggesting that PINK1 and Parkin might regulate mitochondrial dynamics by interacting with the mitochondrial fusion/fission machinery [8]–[10].

PINK1 and Parkin have also been suggested to collaborate to form a mitochondrial quality control system [11], [12]. Despite being mainly cytosolic under normal conditions, Parkin can be mobilized to damaged mitochondria that have decreased membrane potential [11]. This translocation of Parkin requires the function of PINK1, which is stabilized and accumulates on damaged mitochondria [12]. Parkin recruited to damaged mitochondria can further ubiquitinate mitochondrial proteins to mark the damaged mitochondria for degradation by autophagy [11], [13], [14]. These studies offered an attractive molecular mechanism linking the inactivation of PINK1 or Parkin to the accumulation of dysfunctional mitochondria. However, most of these studies were carried out in cell culture. Their *in vivo* relevance remains to be determined.

The target of rapamycin (TOR) protein is an evolutionarily conserved serine/threonine protein kinase that functions as a master regulator of many crucial cellular processes, including protein translation, mRNA transcription, autophagy and cytoskeletal organization [15]. TOR exerts its regulatory function by integrating diverse cues ranging from extracellular growth factors to intracellular levels of ATP, amino acids and oxygen [15]. In response to ATP depletion, for example, TOR signaling is suppressed by the AMP-activated protein kinase (AMPK), leading to subsequent inhibition of S6 kinase (S6K)-mediated protein translation and activation of Atg1-mediated autophagy [16]–[18].

In an effort to further understand the mechanisms of PINK1 and Parkin pathogenesis, we performed genetic screens to find modifiers of *PINK1* mutant phenotypes. We identified S6K and Atg1 as strong modifiers of PINK1. We found that activated S6K acts through protein translational regulation to significantly enhance muscle degeneration and DA neuron loss in *PINK1* mutant. Together with our previous study of LRRK2 [19], this result supports that impaired translational control is an integral part of PD pathogenesis. We also found that Atg1 OE could suppress *PINK1* mutant phenotypes even in the presence of constitutively active S6K, and the rescuing effect of Atg1 OE was dependent on its ability to directly promote autophagy, suggesting that enhancing autophagy represents another efficient way to combat PINK1-related Parkinson's disease. Since reduced S6K activation and enhanced autophagy were already observed in the *PINK1* mutant background, the protective effects observed after further strengthening these processes suggest that they represent compensatory responses to PINK1 inactivation. Pharmacological augmentation of these responses thus holds significant therapeutic value.

## Results

### Gain- and loss-of-function analyses reveal strong genetic interactions between PINK1 and the TOR and autophagy pathways

To better understand PINK1 pathogenesis, we performed both gain-of-function and loss-of-function genetic screens to find genetic enhancers and suppressors of *PINK1* mutant phenotypes. *PINK1* mutant flies exhibit an easily observable abnormal wing posture (either held-up or drooped) caused by indirect flight muscle degeneration. Therefore, we used the *Mhc-Gal4* driver to direct the expression of *UAS-PINK1 RNAi* transgenes specifically in the muscle and used the penetrance of the abnormal wing posture phenotype as an indicator of genetic interaction in our screens. *PINK1 RNAi* line, which exhibited weaker wing posture phenotype than the *PINK1* null mutant, allowed us to screen for both enhancers and suppressors in the same screens by scoring the percentage of flies exhibiting abnormal wing posture at young and old ages.

**Figure 1:**
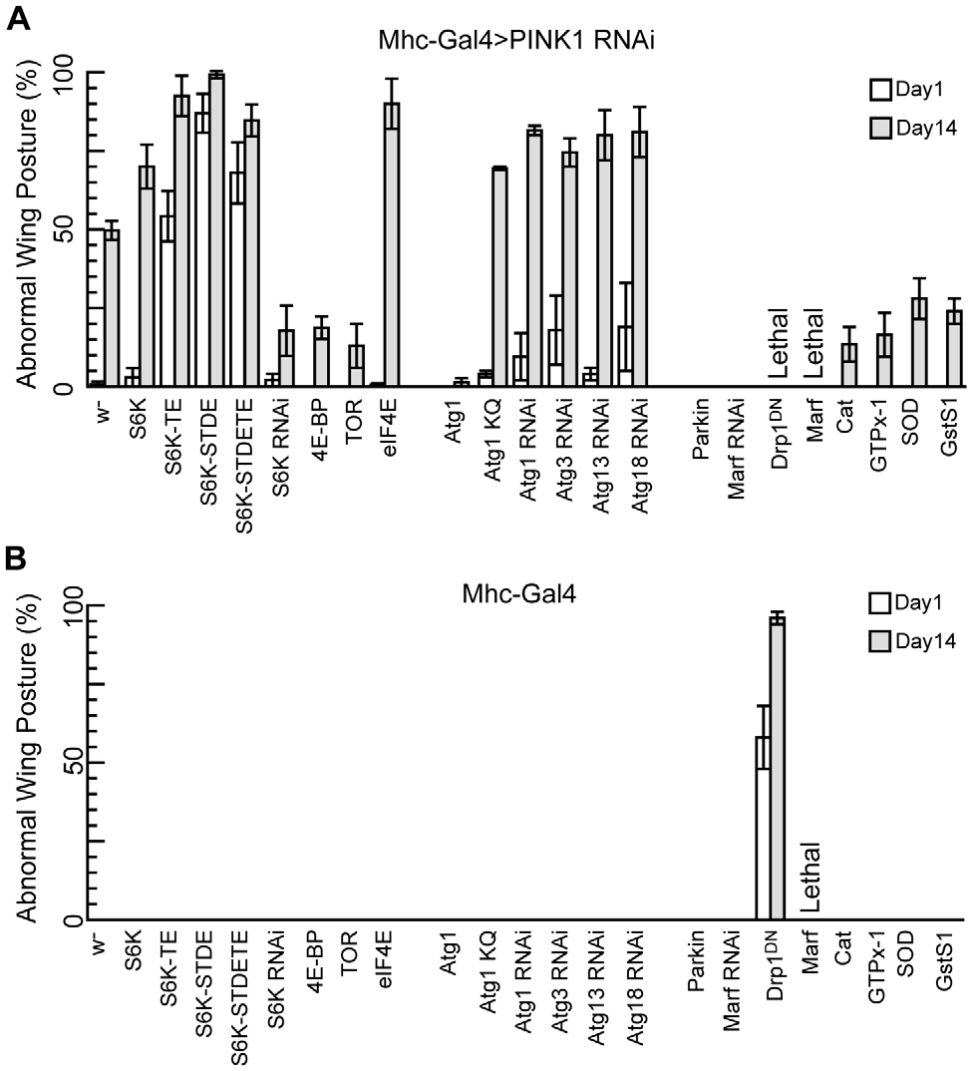
S6K and Atg1 act as genetic modifiers of *PINK1* RNAi. The flies of each indicated genotype were crossed to *Mhc-Gal4>PINK1 RNAi* flies (A) or *Mhc-Gal4* flies (B), and the percentage of male offspring with abnormal wing posture phenotype was scored at 1-day and 14-day after eclosion. The flies were aged at 29°C. Data are presented as mean ± s.e.m. The genetic interactions between *PINK1* and genes of the TOR pathway, autophagy pathway, mitochondrial fusion and fission machinery or antioxidant genes are demonstrated. The differences in abnormal wing posture phenotype between the genetic interaction flies and control flies shown in (A) are all statistically significant (*P*<0.005 in Student's *t*-test).

In our genetic screens, we first took an unbiased approach by screening a collection of ∼300 EP lines, and based on the information obtained from this unbiased screen we implemented a more targeted approach by screening genes involved in specific pathways or processes. In our unbiased screen, we uncovered ∼30 lines that either enhanced or suppressed *PINK1* RNAi phenotype (data not shown). These lines are associated with genes that have diverse functions, suggesting that PINK1 may functionally interact with many different cellular pathways. The strongest modifiers of *PINK1* RNAi phenotype were identified in our targeted screen and one of the strongest enhancers of *PINK1* RNAi-induced abnormal wing posture is S6 kinase (S6K). S6K is one of the downstream effectors of TOR, a master regulator of cell growth and proliferation. In response to cellular growth stimuli, TOR phosphorylates S6K to upregulate the synthesis of ribosomal proteins and translation initiation and elongation factors [20]. When wild type (WT) S6K was overexpressed in the muscle of *PINK1 RNAi* flies, the penetrance of the abnormal wing posture phenotype was greatly enhanced in an age-dependent manner (Figure 1A). Even more dramatic enhancement of the abnormal wing posture was observed when S6K-TE, S6K-STDE, or S6K-STDETE, the phosphomimetic, constitutively active forms of S6K [21], were co-expressed (Figure 1A). In these cases, more than 50% of the flies had abnormal wing posture at 1-day old, whereas virtually none of the *PINK1 RNAi* flies of the same age showed the phenotype. The phenotype became stronger in 14-day-old flies. Overexpression of WT or constitutively active S6Ks in wild type background did not affect wing posture, even after the flies were aged for weeks, suggesting that the effect on wing posture caused by S6K was specific to the *PINK1 RNAi* background (Figure 1B). On the other hand, when we reduced S6K function through *S6K* RNAi, it effectively attenuated *PINK1* RNAi effects (Figure 1A). Similar genetic interaction between PINK1 and constitutively active S6K was observed in *PINK^B9^* mutant background (Figure S1). This result, together with the observation that S6K OE did not affect PINK1 protein level (Figure S2), suggested that the genetic interaction between PINK1 and S6K was not due to a possible regulation of PINK1 expression by S6K.

The stronger effects of the phosphomimetic, constitutively active forms of S6K than WT S6K is consistent with the fact that S6K function is tightly controlled by TOR through ordered phosphorylation of multiple Ser/Thr residues [22], [23]. As S6K is one of the downstream effectors of TOR that regulate protein translation, we further tested whether other components of the TOR pathway that regulate translation also interact with PINK1 genetically. The eukaryotic translation initiation factor 4E (eIF4E)/eIF4E-binding protein (4E-BP) axis of translational control is also regulated by TOR. TOR signaling leads to 4E-BP phosphorylation, weakening its binding to eIF4E and releasing its inhibition on translational initiation [24]. Reducing translation by 4E-BP OE mildly suppressed the abnormal wing posture phenotype in *PINK1 RNAi* background. Conversely, eIF4E OE greatly enhanced the abnormal wing posture phenotype in aged *PINK1 RNAi* flies. However, these flies at 1-day old showed relatively normal wing posture, indicating that the effect of eIF4E is milder compared to constitutively active S6K. Consistent with the above genetic interactions, dTOR OE, which phenocopies *dTOR* loss-of-function effects in *Drosophila* [25], also suppressed *PINK1* RNAi phenotypes. Together, these results support a strong functional interaction between PINK1 and TOR-mediated translational regulation.

In addition to enhancers, we also recovered strong suppressors of *PINK1* RNAi phenotypes, with Atg1 being one of them. Atg1 is a kinase that has been suggested to play an essential role in the initiation of autophagy. Previous studies have shown that Atg1 OE in *Drosophila* fat body was sufficient to induce autophagy, and high level of Atg1 expression could cause growth arrest and apoptosis [26]. When we expressed high level of Atg1 in the muscle using the *Mhc-Gal4* driver and strong *UAS-Atg1* transgenes, the flies either failed to eclose or showed strong abnormal wing posture, possibly due to excess apoptosis in the flight muscle (data not shown). However, when we used a weaker Atg1 OE line (*UAS-Atg1^GS10797^*) [26], the flies enclosed with normal wing posture and flight ability (Figure 1B, data not shown). Interestingly, this mild overexpression of Atg1 completely suppressed the abnormal wing posture caused by PINK1 knockdown (Figure 1A), which was not due to a change in PINK1 expression level (Figure S2). Conversely, *PINK1* RNAi phenotypes were exacerbated by the overexpression of *Atg1* RNAi or *Atg1^K38Q^*, a kinase-dead form of Atg1 that acted in a dominant-negative fashion [26]. Similarly, RNAi-mediated knockdown of *Atg3*, *Atg13* and *Atg18*, all of which are essential for autophagy in *Drosophila* [27], [28], also enhanced the abnormal wing posture phenotype in *PINK1 RNAi* background (Figure 1A). These data indicate that autophagy is physiologically relevant to PINK1 pathogenesis and that mild induction of autophagy protects against PINK1 pathogenesis.

To further validate the effectiveness of our screen, we tested genes that were known to genetically interact with PINK1. We found that Parkin OE and *Marf* RNAi could completely suppress the *PINK1* RNAi phenotypes (Figure 1A), consistent with previous findings [5]–[10]. In contrast to the rescuing effect of *Marf* RNAi, overexpression of Marf alone in the muscle, which would lead to excessive mitochondrial fusion, caused lethality at third instar larval stage (Figure 1B). Flies overexpressing a dominant-negative form of Drp1 (Drp1^DN^), which inhibited mitochondrial fission, were viable but showed strong abnormal wing posture phenotype (Figure 1B). When Drp1^DN^ was expressed in *PINK1 RNAi* background using the *Mhc-Gal4* driver, a synthetic lethality phenotype was observed (Figure 1A). These results are consistent with previously reported genetic interactions between PINK1 and the mitochondrial fusion/fission machinery [8]-[10]. In addition, we tested the effects of antioxidant genes, some of which have been shown to rescue *PINK1* and *Parkin* mutant phenotypes [29], [30]. All four antioxidant genes tested, *catalase (Cat)*, *glutathione peroxidase homolog with thioredoxin peroxidase activity* (*GTPx-1*), *Glutathione S transferase S1 (GstS1)*, and *superoxide dismutase (SOD),* could partially rescue the abnormal wing posture in *PINK1 RNAi* background when overexpressed. However, they were less effective compared to Atg1 and Parkin OE or Marf knockdown (Figure 1A).

### Activated S6K enhances muscle degeneration and DA neuron loss in PINK1 deficiency flies

**Figure 2:**
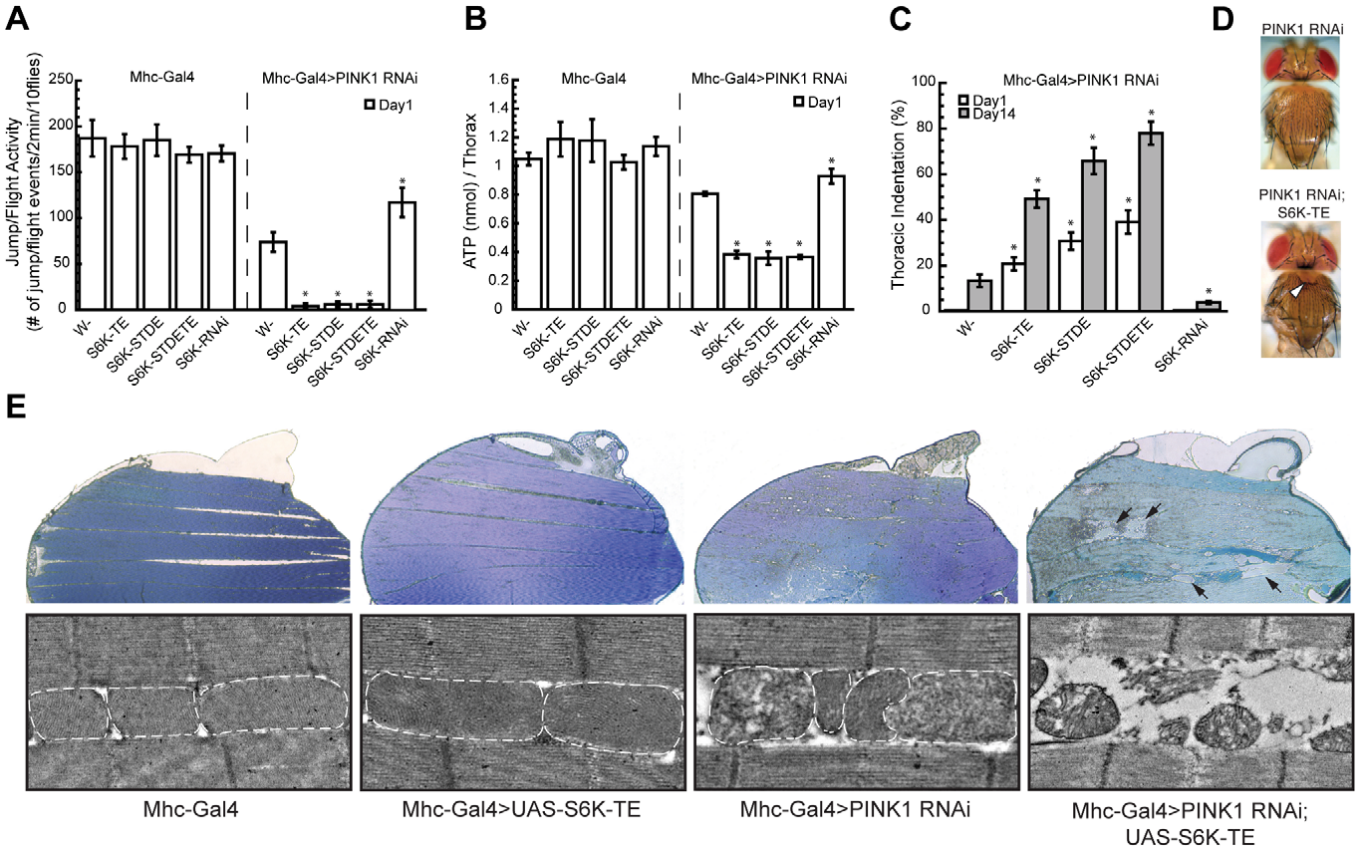
Overexpression or knockdown of S6K strongly modifies *PINK1* RNAi phenotypes in the muscle. Overexpression of constitutively active S6Ks (S6K-TE, S6K-STDE and S6K-STDETE) in the muscle of *PINK1 RNAi* flies completely abolished their jump/flight ability (A), significantly decreased their muscle ATP level (B) and dramatically increased their thoracic indentation (C). In contrast, the overexpression of *S6K RNAi* transgene partially rescued these phenotypes in *PINK1 RNAi* flies. Open and closed bars represented data scored on Day 1 and Day 14 after eclosion, respectively. Data are presented as mean ± s.e.m. Significance was determined by Student's *t* test (*P<0.005). (D) Representative image of thoracic indentation (indicated by the white arrowhead) of *Mhc-Gal4; PINK1 RNAi; S6K-TE* fly at 1-day old (bottom) compared to the normal thoracic phenotype of *Mhc-Gal4, PINK1 RNAi* fly of the same age (top). (E) Overexpression of constitutively active S6K in *PINK1 RNAi* flies dramatically increased muscle degeneration. Sections from resin-embedded thoraces of 1-day-old adult flies were either stained with toluidine blue to visualize overall muscle structure (top panel) or directly visualized using TEM for mitochondrial morphology (bottom panel). WT flies or flies expressing constitutively active S6K show normal muscle structure with healthy, electron-dense mitochondria. Flies expressing *PINK1 RNAi* transgene had small lesions in the muscle with dysfunctional mitochondria showing broken cristae. Co-expression of S6K-TE in *PINK1 RNAi* flies caused more severe degeneration of mitochondria and muscle fibers, generating large lesions in the muscle that were filled with resin during embedding and are readily recognizable (indicated by black arrows).

In addition to abnormal wing posture, *PINK1* mutant flies typically exhibit enlarged mitochondria, energy depletion, muscle degeneration and DA neuron loss [5], [8]. To better understand the genetic interaction between S6K and PINK1, we tested whether S6K affected these phenotypes as well. In one-day-old flies, *Mhc-Gal4*-directed co-expression of constitutively active forms of S6Ks (S6K-TE, S6K-STDE and S6K-STDETE) in *PINK1 RNAi* background completely abolished their flight ability (Figure 2A), significantly decreased ATP level in the muscle (Figure 2B), and dramatically increased thoracic indentation (Figure 2C, 2D), which all indicate increased muscle degeneration. In contrast, overexpression of a *S6K RNAi* transgene in *PINK1 RNAi* background partially rescued these phenotypes (Figure 2A, 2B, 2C). Overexpression of constitutively active S6K or *S6K RNAi* transgenes in wild type flies had no obvious effect in these assays (Figure 2A, 2B), suggesting that their effects on muscle degeneration were specific to the *PINK1 RNAi* background. We further used transmission electron microscopy (TEM) to examine muscle degeneration in detail. At one-day after eclosion, the thoracic muscle of *PINK1 RNAi* flies showed only small lesions, while large areas devoid of muscle tissues were observed in the thoraces of *PINK1 RNAi* flies expressing constitutively active S6K, supporting the conclusion that S6K enhances the muscle degeneration in *PINK1 RNAi* background (Figure 2E). In comparison, the muscle morphology of flies expressing constitutively active S6K alone appeared indistinguishable from that of wild type flies, with healthy, electron-dense mitochondria laying in between muscle fibers in an organized fashion (Figure 2E).

**Figure 3:**
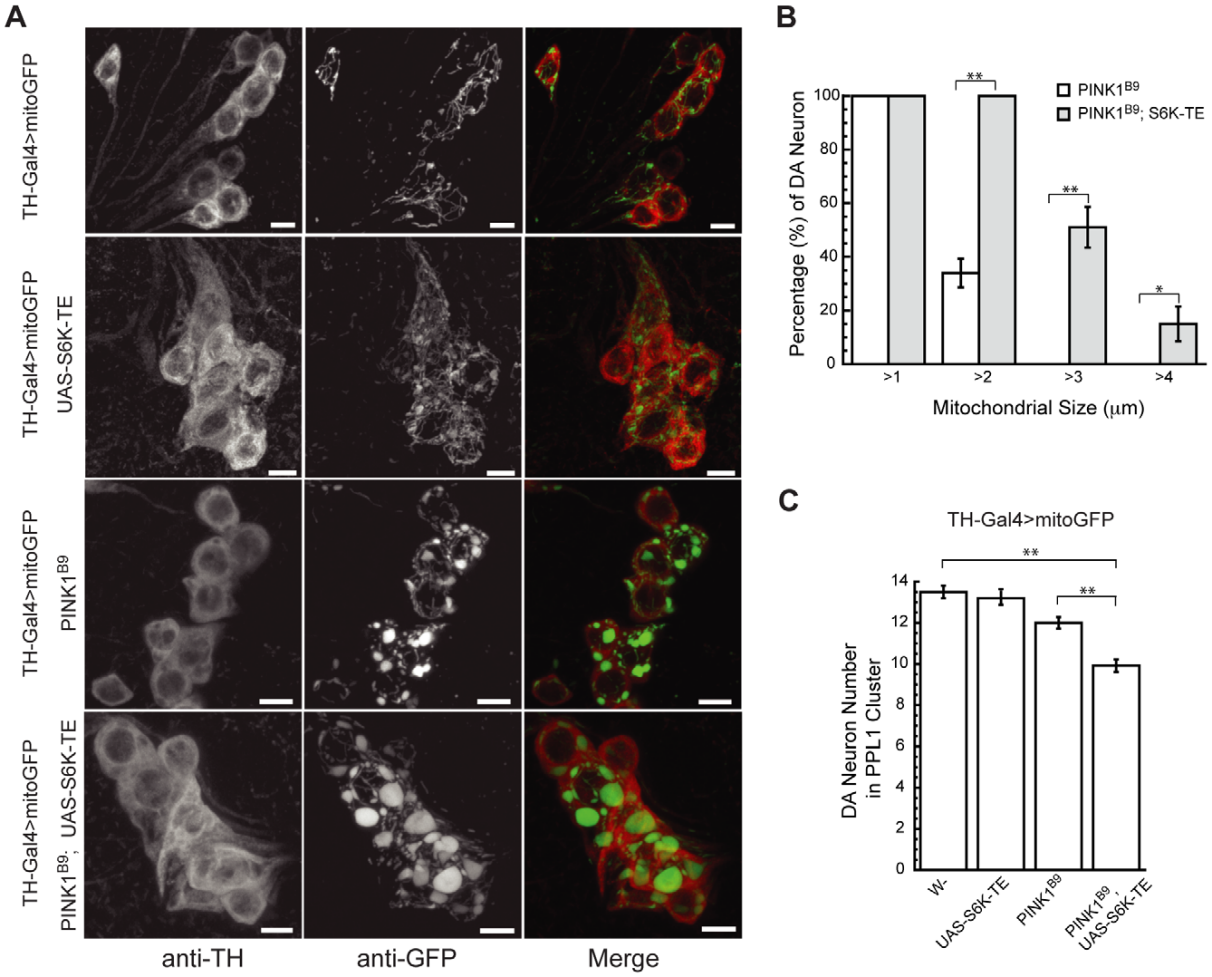
Constitutively active S6K increases mitochondrial aggregation and DA neuron loss in *PINK1* mutants. (A) Overexpression of constitutively active S6K increased the size of swollen or aggregated mitochondria in the DA neurons of *PINK1* mutants. Mitochondrially targeted GFP (mitoGFP) was expressed in the DA neurons using *TH-Gal4* driver [44] to help visualize mitochondrial morphology. Brains of 7-day-old adult flies of the indicated genotypes were immunostained with anti-TH antibody (red) to label DA neuron and anti-GFP antibody (green) to label mitochondria. Images of DA neurons in the PPL1 cluster were shown. Overexpression of S6K-TE in *PINK1* mutant significantly increased the size of mitochondrial aggregates in DA neurons. The scale bar represents 5 µm. (B) Comparison of mitochondrial size distribution in *PINK1* mutants with or without S6K-TE overexpression. Statistical significance was determined by Student's *t* test (**P<0.001, *P<0.05). (C) Overexpression of constitutively active S6K increased DA neuron loss in the PPL1 cluster of *PINK1* mutant. DA neuron number was scored in flies aged for 14 days at 25°C. At least 7 flies were used for each genotype. Statistical significance was determined by Student's *t* test (**P<0.001). Data are presented as mean ± s.e.m.

We further examined mitochondrial morphology in DA neurons using a mitochondrially targeted GFP (mitoGFP) as a marker. When we used the *tyrosine hydroxylase (TH)-Gal4* driver to induce the expression of mitoGFP in wild type DA neurons, most mitochondria exhibited tubular-shaped mitochondrial network (Figure 3A). Overexpression of constitutively active S6K-TE did not change the overall mitochondrial shape, although mitochondrial content appeared increased. *PINK1* mutant (*PINK1^B9^*) showed enlarged mitochondria in DA neurons (Figure 3A), consistent with previous reports [6], [8]. When S6K-TE was expressed in *PINK1^B9^* background, mitochondrial sizes were further increased, frequently doubling those in the *PINK1* mutant in diameter (Figure 3A, 3B). As enlarged mitochondria or mitochondrial clusters are hallmarks of PINK1-related parkinsonism, this further increase of mitochondrial size after S6K-TE overexpression, which is possibly the consequence of inefficient mitophagy (see Discussion and Figure S3), suggests exacerbation of the disease process. Consistent with this idea, the expression of S6K-TE in *PINK1^B9^* mutant background also promoted DA neuron death, as the number of DA neurons in the protocerebral posterior lateral 1 (PPL1) cluster was further decreased compared to *PINK1^B9^* mutants in aged flies (Figure 3C). In summary, the overexpression of constitutively active S6K in *PINK1* mutants significantly enhanced muscle degeneration and DA neuron loss.

### S6K modifies *PINK1* mutant phenotypes through its regulation of translation

**Figure 4:**
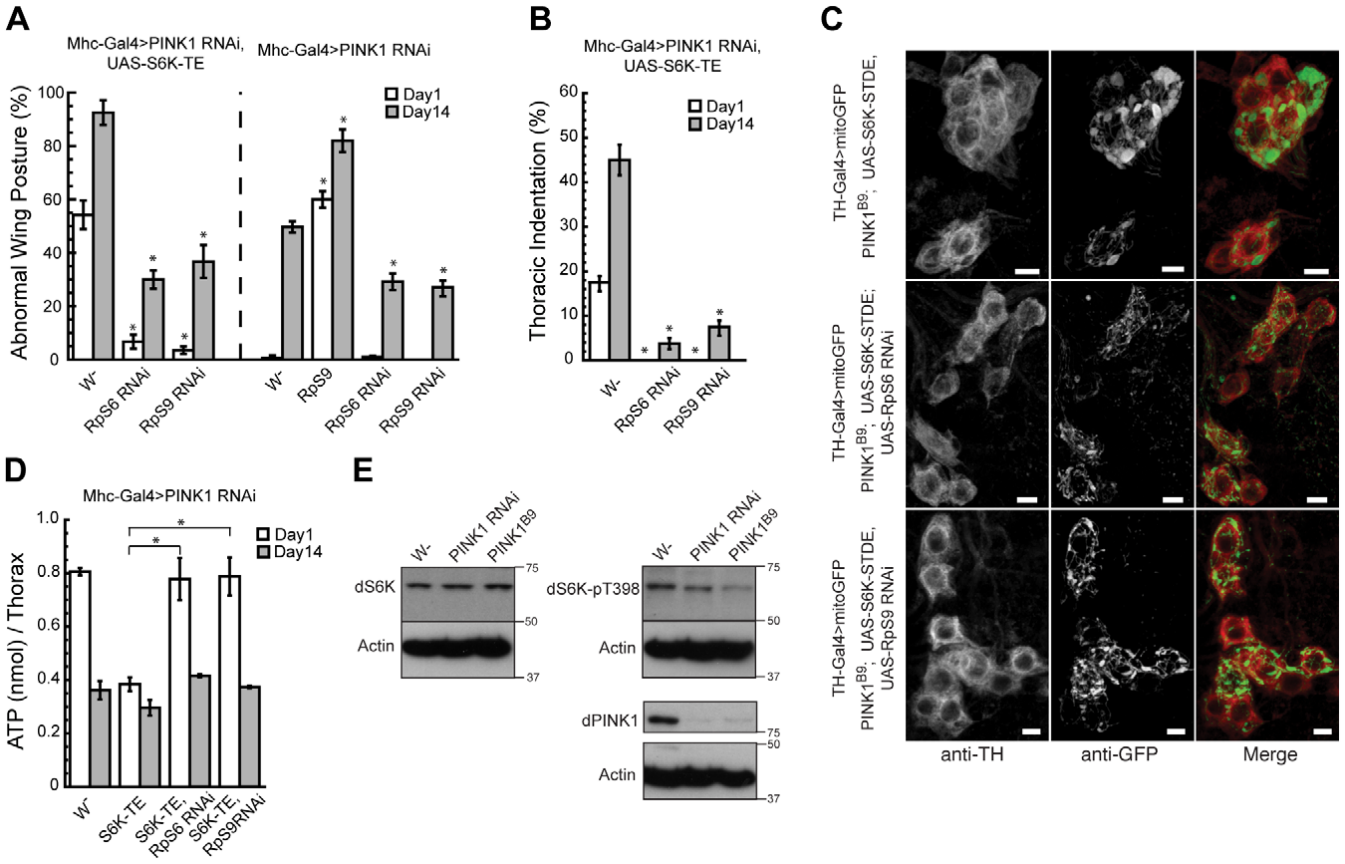
*RpS6* or *RpS9* RNAi blocks the enhancing effects of S6K-TE in *PINK1 RNAi* background. Overexpression of *RpS6* or *RpS9 RNAi* transgenes in *PINK1 RNAi* or *PINK1 RNAi/UAS-S6K-TE* flies efficiently rescued the abnormal wing posture (A), thoracic indentation (B), and energy depletion (D) phenotypes in 1-day-old flies and partially suppressed these phenotypes in 14-day-old flies. Data are presented as mean ± s.e.m. Statistical significance was determined by Student's *t* test (*P<0.001). (C) *RpS6* or *RpS9* RNAi blocked increased mitochondrial aggregation in *PINK1 RNAi, UAS-S6K-TE* flies. The scale bar represents 5 µm. (E) Western blot analysis comparing the levels of dS6K and phosphorylated S6K (T398) in wild type, *Mhc-Gal4>PINK1 RNAi* and *PINK1^B9^* mutant flies. The phosphorylation of S6K was significantly decreased in *PINK1 RNAi* or mutant flies.

Although S6 kinase has been shown to have multiple substrates [31], its best-known function is to phosphorylate the 40S ribosomal subunit S6 (RpS6) and upregulate the translation of proteins involved in ribosomal biogenesis and protein synthesis [20]. To directly examine whether S6K enhances *PINK1* mutant phenotype through increasing translation, we expressed an *RpS6* RNAi transgene together with S6K-TE in the *PINK1 RNAi* background. Significantly, *RpS6* RNAi efficiently blocked S6K-TE's enhancing effect on *PINK1* RNAi-induced abnormal wing posture (Figure 4A), thoracic indentation (Figure 4B), increased mitochondrial aggregation (Figure 4C) and ATP depletion in the muscle (Figure 4D). *RpS6* RNAi also partially suppressed such phenotypes in *PINK1 RNAi* background without S6K-TE co-expression (Figure 4A, data not shown). The effects of *RpS6* RNAi were more obvious in young flies. In aged flies, the rescuing effect of *RpS6* RNAi was mild in both the *PINK1 RNAi* and *PINK1 RNAi/S6K-TE OE* backgrounds (Figure 4A, 4D, data not shown). These results suggested that S6K did act through RpS6 to genetically interact with PINK1; however, reduction of translation through *RpS6* RNAi only provided partial suppression of *PINK1* RNAi phenotypes. This could be due to either inefficient knockdown of *RpS6* function by RNAi or the involvement of other pathogenic pathway(s).

To further confirm the genetic interaction between PINK1 and the protein translational control pathway, we screened more than 20 EP lines expressing cytosolic or mitochondrial ribosomal subunits to see if any of these lines could also modify *PINK1* RNAi phenotypes. Interestingly, one line that overexpresses ribosomal protein S9 (RpS9) greatly enhanced the *PINK1* RNAi phenotypes (Figure 4A). Further, *RpS9* RNAi was as effective as *RpS6* RNAi in blocking S6K-TE's enhancing effects on *PINK1* RNAi phenotypes (Figure 4A–4D). Previously, RpS9 knockdown was shown to significantly reduce the rate of protein synthesis and cell proliferation in primary human fibroblasts and tumor cell lines [32]. It is therefore likely that *RpS9* RNAi mitigates the effects of constitutively active S6Ks through downregulating translation.

To test whether S6K and the related translational control pathway is normally involved in PINK1 pathogenesis, we examined the levels of S6K and phosphorylated S6K in *PINK1* mutants. We found that the level of total S6K in *PINK1 RNAi* or *PINK1^B9^* mutant flies was largely unchanged. However, the level of phosphorylated, active form of S6K was significantly decreased (Figure 4E), suggesting that there is decreased TOR signaling and protein translation in *PINK1* mutant background. Since protein translation is a very energy-consuming process, reduction of translation could serve as a compensatory response to the mitochondrial dysfunction caused by PINK1 inactivation.

### Atg1 activation partially rescues muscle degeneration in *PINK1* mutant

**Figure 5:**
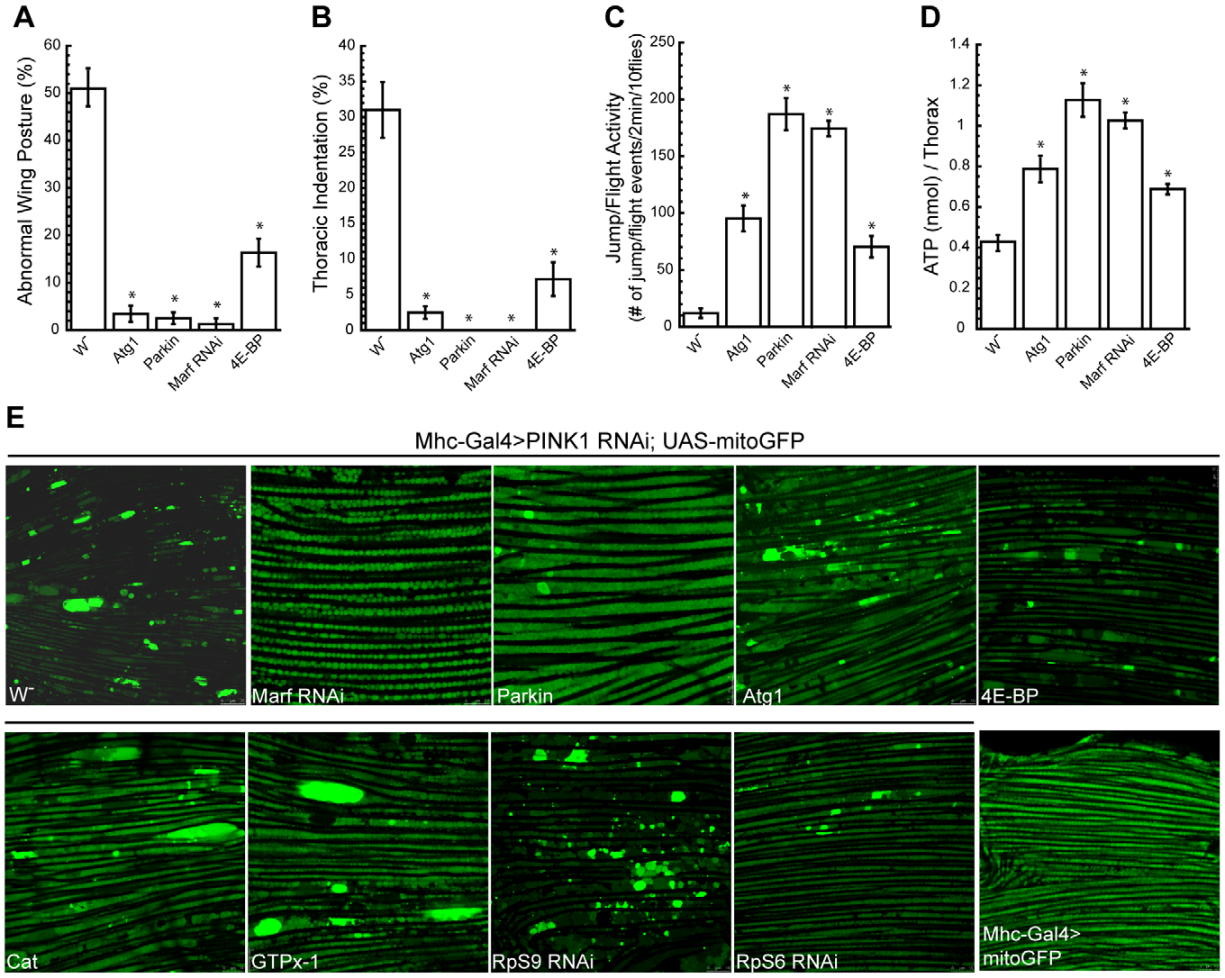
Overexpression of Atg1 rescues *PINK1* mutant phenotypes. Overexpression of Atg1 in the muscle of *PINK1^B9^* mutants rescued their abnormal wing posture (A), thoracic indentation (B), jump/flight activity (C) and muscle ATP level (D). Data are presented as mean ± s.e.m. Statistical significance was determined by Student's *t* test (*P<0.001). (E) Atg1 overexpression did not completely rescue the mitochondrial aggregation phenotype in the muscle of *PINK1 RNAi* flies. mitoGFP was expressed in the muscle using *Mhc-Gal4* driver to visualize mitochondrial morphology by live imaging. Wild type flies showed mitochondria of relatively uniform sizes (bottom right), while *PINK1 RNAi* flies had bright mitochondrial aggregates. Only the co-expression of Marf RNAi or Parkin OE was able to efficiently rescue the mitochondrial aggregation phenotype in the *PINK1* RNAi background.

As mentioned earlier, Atg1 emerged as a strong suppressor of *PINK1* RNAi phenotype in our screen. To confirm this result, we tested the effect of Atg1 OE in *PINK1^B9^* mutant, which exhibited stronger phenotypes than *PINK1 RNAi* flies. Similar to the results obtained using *PINK1 RNAi* flies, Atg1 OE could efficiently suppress the abnormal wing posture and thoracic indentation phenotypes of *PINK1^B9^* flies (Figure 5A, 5B). Of note, our behavior test and muscle ATP level analysis indicated that the rescuing effect of Atg1 OE was not as strong as Parkin OE or *Marf* RNAi (Figure 5C, 5D). When we used mitoGFP to examine mitochondrial morphology in the muscle, Parkin OE and *Marf* RNAi could almost completely suppress the mitochondrial aggregation phenotype, but we could still see enlarged mitochondria in *PINK1* mutant overexpressing Atg1 (Figure 5E).

Similar to Atg1 OE, 4E-BP OE only partially rescued *PINK1^B9^* mutant phenotypes (Figure 5A–5D). The overexpression of *Catalase*, *GTPx-1*, *RpS6* RNAi or *RpS9* RNAi also could not fully rescue the abnormal mitochondrial morphology phenotype in the *PINK1* mutant (Figure 5E). Therefore, these genes modified *PINK1* mutant phenotypes without effectively rescuing mitochondrial morphology, suggesting that they might act downstream or in parallel to the mitochondrial dynamics pathway.

### *Atg1* OE rescues *PINK1* mutant phenotype by inducing autophagy

**Figure 6:**
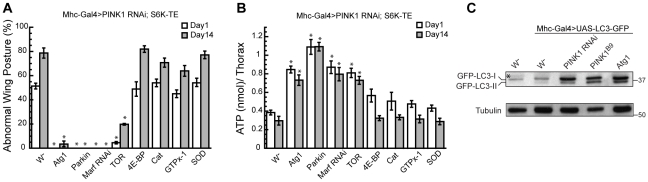
Atg1 OE rescues *PINK1* RNAi phenotype by inducing autophagy. (A, B) The rescuing effect of Atg1 OE in *PINK1 RNAi* flies was not dependent on S6K inhibition. Atg1 OE, as well as Parkin OE or *Marf* RNAi, efficiently rescued the abnormal wing posture (A) and muscle energy depletion (B) in *PINK1 RNAi/S6K-TE* flies. In contrast, overexpression of 4E-BP and the antioxidant genes were not as effective. Data are presented as mean ± s.e.m. Statistical significance was determined by Student's *t* test (*P<0.001). (C) Overexpression of Atg1 was sufficient to induce autophagy in fly muscle. *UAS-LC3-GFP* was expressed in the muscle of flies with the indicated genetic background, and the level of autophagy was determined by Western Blot using anti-GFP antibody. Overexpression of Atg1 significantly increased the level of LC3-II in the muscle, indicating increased autophagy. Increased autophagy was also observed in *PINK1 RNAi* and *PINK1* mutant flies. (* indicates a cross-reaction band).

Atg1 is a Ser/Thr protein kinase involved in the initiation of autophagosome formation, which is under the control of TOR signaling. The loss of TOR signaling promotes the association of Atg1 with Atg13 and Atg17, which further recruit other Atg proteins to the pre-autophagosomal structure to mediate the formation of autophagosome [33]. In *Drosophila*, Atg1 OE alone is sufficient to induce autophagy in the fat body [26]. In addition to being a downstream effector of TOR, Atg1 can also exert feedback inhibitory effect on TOR. Atg1 OE has been shown to cause reduced phosphorylation of *Drosophila* S6K (dS6K) at T398, indicating downregulation of TOR signaling by Atg1 [26], [34]. Since S6K OE and Atg1 OE exert opposite effects in *PINK1 RNAi* background, we next tried to distinguish whether the rescuing effect of Atg1 OE was due to the inhibition of S6K function or induction of autophagy. To test whether Atg1 suppressed *PINK1* RNAi-induced abnormal wing posture through inhibition of S6K, we expressed Atg1 together with S6K-TE in the *PINK1 RNAi* background. S6K-TE harbors the T398E mutation that mimics the phosphorylated form of S6K, which is constitutively active and cannot be suppressed by Atg1 [21]. Strikingly, Atg1 OE could strongly rescue the abnormal wing posture and ATP depletion phenotypes in the *PINK1 RNAi/S6K-TE OE* background (Figure 6A, 6B), suggesting that the rescuing effect of Atg1 did not rely on its known effect on S6K phosphorylation. Similarly, Parkin OE or *Marf* RNAi also significantly suppressed the abnormal wing posture and energy depletion phenotypes in *PINK1 RNAi/S6K-TE OE* background (Figure 6A, 6B). In contrast, overexpression of *4E-BP* or the anti-oxidant genes, such as *GTPx-1*, *Cat* and *SOD*, were not as effective (Figure 6A, 6B).

To test whether Atg1 OE rescued *PINK1* RNAi phenotype by inducing autophagy, we first tested whether Atg1 OE could directly induce autophagy in the muscle, as observed in the fat body [26]. We used LC3-GFP as a marker to examine the lipidation of LC3 in different genetic backgrounds and used GFP antibody to detect mobility shift of LC3-GFP. Compared to the control, Atg1 OE led to an increased level of LC3-II, indicating induction of autophagy (Figure 6C). To test whether PINK1 deficiency affects autophagy *in vivo*, we introduced LC3-GFP into *PINK1 RNAi* and *PINK1^B9^* backgrounds. Elevated autophagy was observed in both cases (Figure 6C). Thus, autophagy is basally induced in *PINK1* mutant and further enhancement of autophagy is protective, suggesting that similar to decreased protein translation, increased autophagy also represents a compensatory response in PINK1 loss-of-function background.

**Figure 7:**
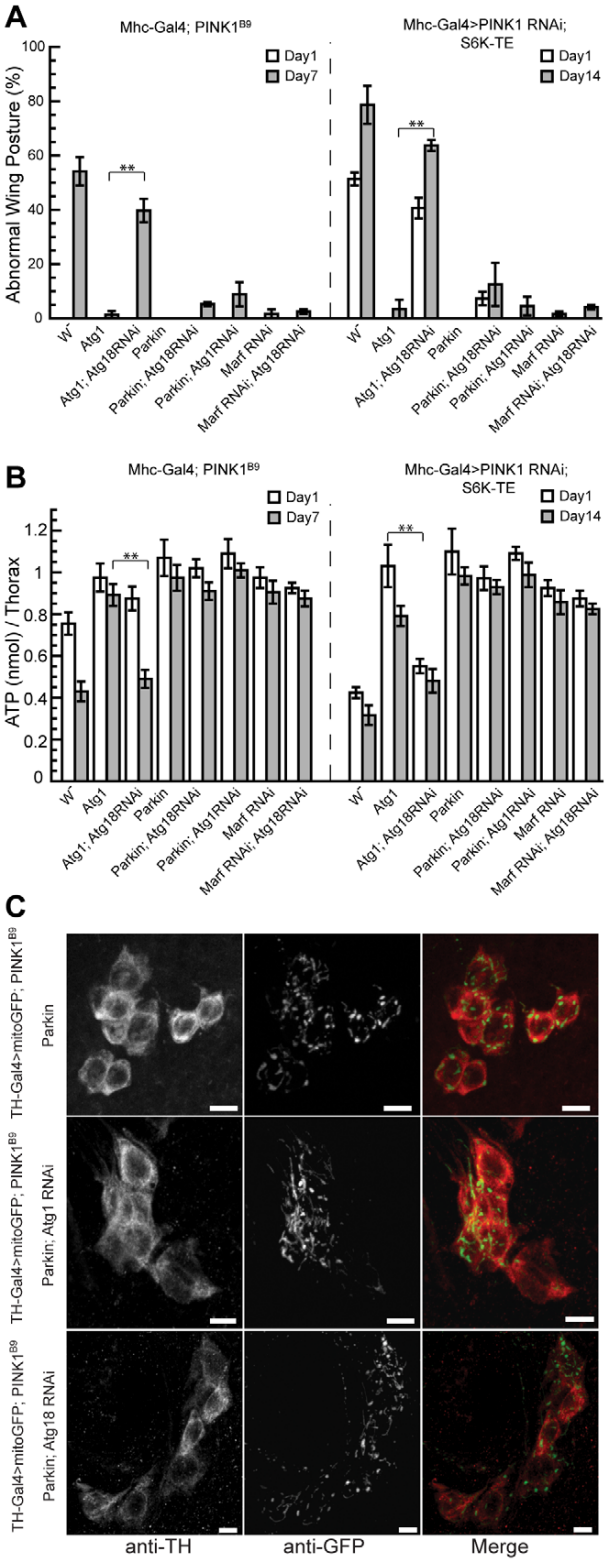
*Atg18* RNAi blocks the rescuing effect of Atg1 OE but not that of Parkin OE or *Marf* RNAi. The rescuing effect of Atg1 OE on the abnormal wing posture (A) and muscle energy depletion (B) phenotypes could be blocked by the co-expression of *Atg18* RNAi, suggesting that Atg1 functions through inducing autophagy to rescue *PINK1* RNAi phenotype. In contrast, the rescue of *PINK1* RNAi phenotypes by Parkin OE or *Marf* RNAi were largely unaffected by the disruption of Atg1 or Atg18 through RNAi. The tests were carried out in both *PINK1^B9^* mutant and *Mhc-Gal4>PINK1 RNAi/S6K-TE* backgrounds. Data are presented as mean ± s.e.m. Statistical significance was determined by Student's *t* test (**P<0.001). (C) *Atg1* or *Atg18* RNAi did not abolish the rescuing effect of Parkin OE in DA neurons. mitoGFP was expressed in DA neurons using the *TH-Gal4* driver to visualize mitochondrial morphology. Overexpression of Parkin efficiently rescued the mitochondrial aggregation phenotype in *PINK1* mutant (top panel). Similar rescuing effect was observed when *Atg1* RNAi or *Atg18* RNAi was co-expressed with Parkin (middle and bottom panels). The scale bar represents 5 µm.

To further prove that Atg1 OE-induced autophagy was critical for the suppression of *PINK1* RNAi phenotype, we attempted to block the Atg1 OE effects with *Atg18* RNAi. The co-expression of Atg18 RNAi could largely abolish the rescuing effects of Atg1 OE in *PINK1^B9^* mutant or *PINK1 RNAi/S6K-TE OE* backgrounds (Figure 7A, 7B), suggesting that Atg1 OE rescued *PINK1* mutant phenotypes mainly through inducing autophagy.

### Blocking autophagy does not abolish the rescuing effects of Parkin OE or *Marf* RNAi in *PINK1* mutant background

Recent cell culture studies showed that Parkin is specifically recruited to dysfunctional mitochondria to mediate their elimination by the autophagy pathway, and the lack of PINK1 prevented this process, suggesting that Parkin plays an essential role in the selective elimination of damaged mitochondria [11]. We sought to directly test the role of autophagy in Parkin's rescue of *PINK1* mutant phenotypes *in vivo*. In stark contrast to the efficient blockage of Atg1's rescuing effect by *Atg18* RNAi, *Atg18* RNAi failed to block Parkin's rescue of the abnormal wing posture (Figure 7A) and energy depletion (Figure 7B) phenotypes in *PINK1^B9^* and *PINK1 RNAi/S6K-TE OE* flies. Similarly, *Atg1* RNAi also could not block the rescuing effect of Parkin (Figure 7A, 7B). Consistent with these results obtained in the muscle, *Atg1* RNAi or *Atg18* RNAi failed to block the ability of Parkin to rescue the mitochondrial morphology phenotype in *PINK1* mutant DA neurons (Figure 7C). These results suggest that Parkin might act through other mechanisms to rescue *PINK1* mutant phenotype than solely promoting selective autophagy. *Atg18* RNAi also failed to block the rescuing effects of *Marf* RNAi in *PINK1^B9^* and *PINK1 RNAi/S6K-TE OE* backgrounds (Figure 7A, 7B), suggesting that decreased mitochondrial fusion can rescue *PINK1* mutant phenotype independent of the autophagy pathway.

## Discussion

The occurrence and the progression of Parkinson's disease can be determined by both genetic predisposition and environmental insults. Recent human genetic studies have identified many genes responsible for the heritable forms of the disease, greatly enhancing our understanding of disease pathogenesis [35]. By analyzing the cellular pathways that interact with these genes, hopefully we will ultimately find ways to better understand and treat this devastating disease.

Previously, PINK1 and Parkin have been suggested to interact with mitochondrial fusion/fission machinery and the autophagy pathway [8]–[11]. In this study, we found that PINK1 also genetically interacted with the protein translation pathway. Increased global protein translation with S6K or eIF4E OE exacerbated *PINK1* mutant phenotypes, while decreased translation had the opposite effects. Overexpression of constitutively active S6Ks dramatically enhanced muscle and DA neuron degeneration in *PINK1* mutant flies, which could be mitigated by the co-expression of *RpS6* RNAi or *RpS9* RNAi, supporting that the TOR/S6K pathway modifies *PINK1* mutant phenotypes through regulating global translation. Recently, we have reported that pathogenic leucine-rich repeat kinase 2 (LRRK2), which represents the most frequent molecular lesions found in Parkinson's disease, promotes 4E-BP phosphorylation, resulting in increased eIF4E-mediated translation, enhanced sensitivity to oxidative stress, and DA neuron loss [19]. Taken together, our results support the idea that deregulated protein translation is generally involved in the pathogenesis of Parkinson's disease.

Deregulated translation affects Parkinson's disease pathogenesis most likely at the level of energy metabolism, since protein translation is a very energy-consuming process, of which ribosomal biogenesis is the most costly, consuming approximately 80% of the energy in proliferating cells [36]. Here we show that forced upregulation of ribosomal biogenesis in the fly muscle by the overexpression of constitutively active S6K was well tolerated in WT flies; however, such manipulation in *PINK1 RNAi* flies completely abolished their flight ability, depleted ATP in the muscle and enhanced muscle and DA neuron degeneration. The tolerance of increased protein translation by wild type flies is probably due to the existence of an intact mitochondrial quality control system containing PINK1 and Parkin, which can either eliminate damaged mitochondria generated during elevated energy production or minimize damages caused by increased ROS generated during energy production. However, in *PINK1* or *Parkin* mutants that lack a functional mitochondrial quality control system, increased protein translation and the corresponding energy demand will translate into increased ROS generation, accumulation of dysfunctional mitochondria, and eventual energy depletion and tissue degeneration. Since downregulation of translation through knockdown of *S6K*, *RpS6,* or *RpS9* is beneficial to *PINK1* mutant flies, and S6K activity is already tuned down in *PINK1* mutant flies, reduction of translation likely represents one of the cellular compensatory responses to the energy deficit caused by mitochondrial dysfunction in *PINK1* mutants. Interestingly, partial reduction of S6K activity prolonged fly lifespan, whereas increased S6K activity had the opposite effects on longevity [37]. The effects of S6K on animal lifespan and *PINK1* mutant phenotypes can both be explained by the energy metabolism hypothesis and they offer a tantalizing link between aging and the pathogenesis of Parkinson's disease.

Supporting the energy metabolism model, we show that downregulation of protein translation by knocking down positive regulators of translation (S6K, RpS6, RpS9) or overexpressing a negative regulator (4E-BP) could rescue *PINK1* mutant phenotypes. These manipulations presumably act by preserving cellular energy and reducing the workload and ROS production of mitochondria. Previously, 4E-BP OE was suggested to rescue *PINK1* mutant phenotype by upregulating Cap-independent translation of stress related genes, including antioxidant genes [30], and boosting antioxidant gene activity has been suggested as a therapeutic strategy in the PINK1 and Parkin models of Parkinson's disease [38]. We found that although overexpression of antioxidant genes, such as *Catalase*, *GTPx-1*, *SOD* and *GstS1*, all showed some degree of rescue of *PINK1* mutant phenotypes, their effects were in general weaker than that of *Atg1* OE, *Parkin* OE, or *Marf* RNAi, particularly in the *PINK1 RNAi/S6K-TE OE* background. These data suggest that increasing autophagy and mitochondrial fission might be better choices to combat PINK1-related Parkinson's disease.

Autophagy is a conserved cellular process through which cytoplasmic content or defective intracellular organelles can be eliminated or recycled. Although autophagy is usually induced under adverse conditions to provide means for survival, basal level of autophagy in the cell is just as critical to the physiological health of the organism, since defects in autophagy are frequently associated with cancer, neurodegeneration, and aging [39]. The induction of autophagy leads to the *de novo* formation of double membrane structure called isolation membrane, which expands to form a sealed compartment named autophagosome that will engulf materials destined for degradation. The large size of mitochondria likely poses a challenge for the autophagy machinery, as engulfment of an entire mitochondrion requires a significant amount of building materials for autophagosome formation. This is especially the case in *PINK1* mutant where dysfunctional mitochondria becomes grossly swollen or aggregated. Previously, we and others showed that increased mitochondrial fission or *Parkin* OE could efficiently rescue the enlarged mitochondria phenotype in *PINK1* mutants [5]–[10]. The rescuing effect by increased mitochondrial fission could be due to the fact that it decreases mitochondrial size and makes it easier for the autophagosome to engulf the entire mitochondrion during mitophagy (Figure S3). In addition, increased mitochondrial fission could facilitate the segregation of the healthy part of a mitochondrion from the unhealthy part, thus enhancing the selective elimination of dysfunctional mitochondria through mitophagy [40]. Supporting the mitophagy model, Parkin has been proposed to promote the efficient removal of damaged mitochondria by selectively ubiquitinating proteins on damaged mitochondria [11]. A key prediction of the mitophagy model is that the protective effects of Parkin OE and increased mitochondrial fission as in the case of *Marf* RNAi will depend on the autophagy pathway. Surprisingly, we found that blocking autophagy through *Atg1* RNAi or *Atg18* RNAi failed to block Parkin OE or *Marf* RNAi's rescuing abilities in *PINK1* mutant, although *Atg18* RNAi was effective in blocking the rescuing ability of *Atg1* OE. This result suggests that the rescuing effect of *Parkin* OE or *Marf* RNAi is not entirely dependent on autophagy, and that other processes are likely involved. For example, Parkin has been suggested to promote mitochondrial biogenesis [41] and regulate protein translation [42]. Further studies are needed to elucidate the exact molecular functions of Parkin that are critically involved in mitochondrial function and tissue maintenance *in vivo*.

Given the well-established catabolic role of autophagy in degrading cytoplasmic contents, it helps recycle nutrients and provide energy source needed for survival under harsh conditions. In *PINK1* mutants that suffer energy deficit due to mitochondrial dysfunction, induction of autophagy would present as a compensatory response to cope with the limited energy supply. Indeed, we found that basal autophagy is induced in *PINK1* mutant, and further increase of autophagy through Atg1 OE protects against PINK1 pathogenesis. Thus, decreased translation and increased autophagy both represent compensatory responses in *PINK1* mutant flies, and further augmentation of these responses can effectively protect against the toxic effects of PINK1 inactivation. A previous study in cultured mammalian cells also indicated that autophagy is induced in response to PINK1 inactivation [43]. Thus, the *in vivo* compensatory responses revealed in this study are likely relevant to PINK1 pathogenesis in mammals. Pharmacological interventions that promote these responses offer potential new treatment strategies for Parkinson's disease.

## Materials and Methods

### Fly strains

Flies were raised according to standard procedures at indicated temperatures. *dPINK1* null mutant line *dPINK1^B9^* was a gift from Dr. Jongkeong Chung [6]. The *TH-GAL4* line was a gift from Dr. Serge Birman [44]. *UAS-mitoGFP* line was a gift from Dr. William Saxton. *UAS-Atg1[6A]*, *UAS-Atg1[6B]*, *UAS-Atg1^KQ^*, and *UAS-Atg13 RNAi* lines were gifts from Dr. Thomas Neufeld [26]. *UAS-Atg1^GS10797^* line was a gift from Dr. Eric Baehrecke. *UAS-Marf* line was a gift from Dr. Alex Whitworth. *UAS-PINK1 RNAi* and *UAS-Parkin* were generated as described [5], [45]. *UAS-Atg1 RNAi*, *UAS-Atg3 RNAi*, *UAS-Atg18 RNAi*, *UAS-RpS6 RNAi*, *UAS-RpS9 RNAi*, and *UAS-S6K RNAi* lines were from Vienna *Drosophila* RNAi Center. All the other lines were from Bloomington Stock Center.

### Muscle histology and transmission electron microscopy analysis

Muscle histology with toluidine blue staining and transmission electron microscopy analysis was performed essentially as described [45], except that Epon resin was used for embedding. For mitochondrial morphology analysis using mitoGFP, indirect fly muscle was dissected out in PBS and examined by live imaging.

### Abnormal wing posture and behavior analyses

For abnormal wing posture analysis, male flies were aged at indicated temperature with 20 flies per vial. The abnormal wing posture penetrance was calculated as the percentage of flies with either held-up or drooped wing posture [5]. For each experiment, at least 60 flies were scored for their wing posture phenotype for each genotype. Each experiment was repeated at least three times. For jump/flight ability analyses, 5 to 10 flies were put into each vial. The jump/flight events were counted for two consecutive minutes, during which vials were gently tapped to initiate those events. Data were normalized and represented to reflect the jump/fly activity of 10 animals. Each of these analyses had been repeated at least three times.

### ATP measurement

The thoracic ATP level was measured using a luciferase based bioluminescence assay (ATP Bioluminescence Assay Kit HS II, Roche Applied Science). For each measurement, two thoraces were dissected out (with wings and legs removed) and immediately homogenized in 100 µl lysis buffer. The lysate was boiled for 5 min and cleared by centrifugation at 20,000 g for 1 min. 2.5 µl of cleared lysate was added to 187.5 µl dilution buffer and 10 µl luciferase, and the luminescence was immediately measured using a Lumat LB 9507 tube luminometer (Berthold Technologies). Each reading was converted to the amount of ATP per thorax based on the standard curve generated with ATP standards. At least 3 measurements were made for each genotype.

### Immunohistochemistry and western blot

Whole-mount immunohistochemistry for TH and mitoGFP was performed as described [46]. Rabbit anti-TH antibody (1∶500)[5] and chicken anti-GFP antibody (1∶5000) (Chemicon International) were used. The images of DA neurons of the protocerebral posterior lateral 1 (PPL1) cluster were collected at 0.5 mm steps along Z-axis on a Leica confocal microscope, and all the images shown were generated by Z-stack deconvolution. Western Blot was performed following standard protocol. Rabbit anti-dS6K antibody was a gift from Dr. George Thomas. Rabbit anti-Phospho-S6K (Thr398) antibody was from Cell Signaling Technology, and chicken anti-GFP antibody was purchased from Chemicon International. Rabbit anti-dPINK1 antibody was generated as described [5].

## Supporting Information

Figure S1 S6K genetically interacts with PINK1. The flies of each indicated genotype were crossed to *Mhc-Gal4; PINK1^B9^* flies and the percentage of male offspring with abnormal wing posture phenotype was scored at 1 day and 7 days after eclosion. The flies were aged at 25°C. Data are presented as mean ± s.e.m.
(0.23 MB AI)

Figure S2 Overexpression of S6K or Atg1 does not affect PINK1 protein expression. Western blot analysis was performed to compare the levels of dPINK1 protein in *Mhc-Gal4/+*, *Mhc-Gal4>S6K-TE,* and *Mhc-Gal4>Atg1* flies. Thoraces of flies with the indicated genotypes were dissected out and dissolved in SDS sample buffer. About 0.5 thorax-equivalent of protein extract was used for Western blot analysis. No significant change in dPINK1 expression level was observed in S6K-TE or Atg1 overexpression flies.
(0.08 MB TIF)

Figure S3 Pathways that can affect PINK1 pathogenesis. PINK1 inactivation generally leads to the accumulation of dysfunctional, enlarged mitochondria. (A) Without intervention, those dysfunctional mitochondria continue to generate reactive oxygen species (ROS) and accumulate damages that lead to mitochondrial death, energy depletion and tissue degeneration. Although autophagy is induced as a compensatory response to PINK1 inactivation, this native response may not be strong enough to allow isolation membrane to efficiently engulf the entire damaged mitochondrion. (B) When autophagy is further induced mildly by Atg1 OE, the generation of more isolation membrane may allow the autophagosomes to form around the entire mitochondrion. However, this process may still be inefficient, with some large dysfunctional mitochondria left unremoved. (C) The efficiency of mitophagy may be further increased when enhanced mitochondrial fission breaks the enlarged mitochondria into smaller ones to allow easy access, expansion of isolation membrane and the maturation of autophagosome. Mitochondrial fission may also selectively segregate damaged part of mitochondria from the healthy one, increasing the specificity and efficacy of mitophagy. The increase in mitochondrial fission could be achieved by direct manipulation of mitochondrial fusion/fission machinery, such as knocking down Marf, or by the overexpression of Parkin, which has been shown to reduce Marf protein level through ubiquitination. However, Parkin OE and Marf RNAi likely exert more functions than simply facilitating autophagy in *PINK1* mutant background, since blocking autophagy does not completely eliminate their protective effects. (D) The dysfunctional mitochondrial phenotype due to PINK1 inactivation can also be ameliorated by increasing the expression of antioxidant genes or decreasing protein translation. Antioxidant genes rescue *PINK1* mutant phenotype by direct scavenging of ROS, while decreased protein translation acts through reducing energy demand to curb ROS production.
(1.22 MB TIF)
