# Supplementary figures and images for: Reduction of Protein Translation and Activation of Autophagy Protect against PINK1 Pathogenesis in Drosophila melanogaster
Source: PLoS Genet. 2010 Dec 9;6(12):e1001237. doi: 10.1371/journal.pgen.1001237 (PMC3000346; doi:10.1371/journal.pgen.1001237)

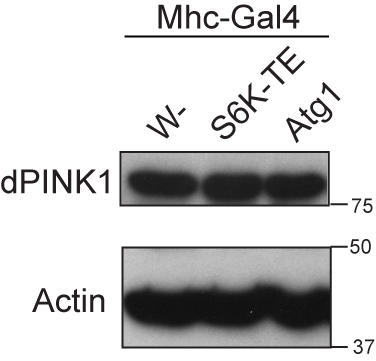

Supplement: Figure S2 — Overexpression of S6K or Atg1 does not affect PINK1 protein expression. Western blot analysis was performed to compare the levels of dPINK1 protein in Mhc-Gal4/+, Mhc-Gal4>S6K-TE, and Mhc-Gal4>Atg1 flies. Thoraces of flies with the indicated genotypes were dissected out and dissolved in SDS sample buffer. About 0.5 thorax-equivalent of protein extract was used for Western blot analysis. No significant change in dPINK1 expression level was observed in S6K-TE or Atg1 overexpression flies. (0.08 MB TIF) [file pgen.1001237.s002.tif]

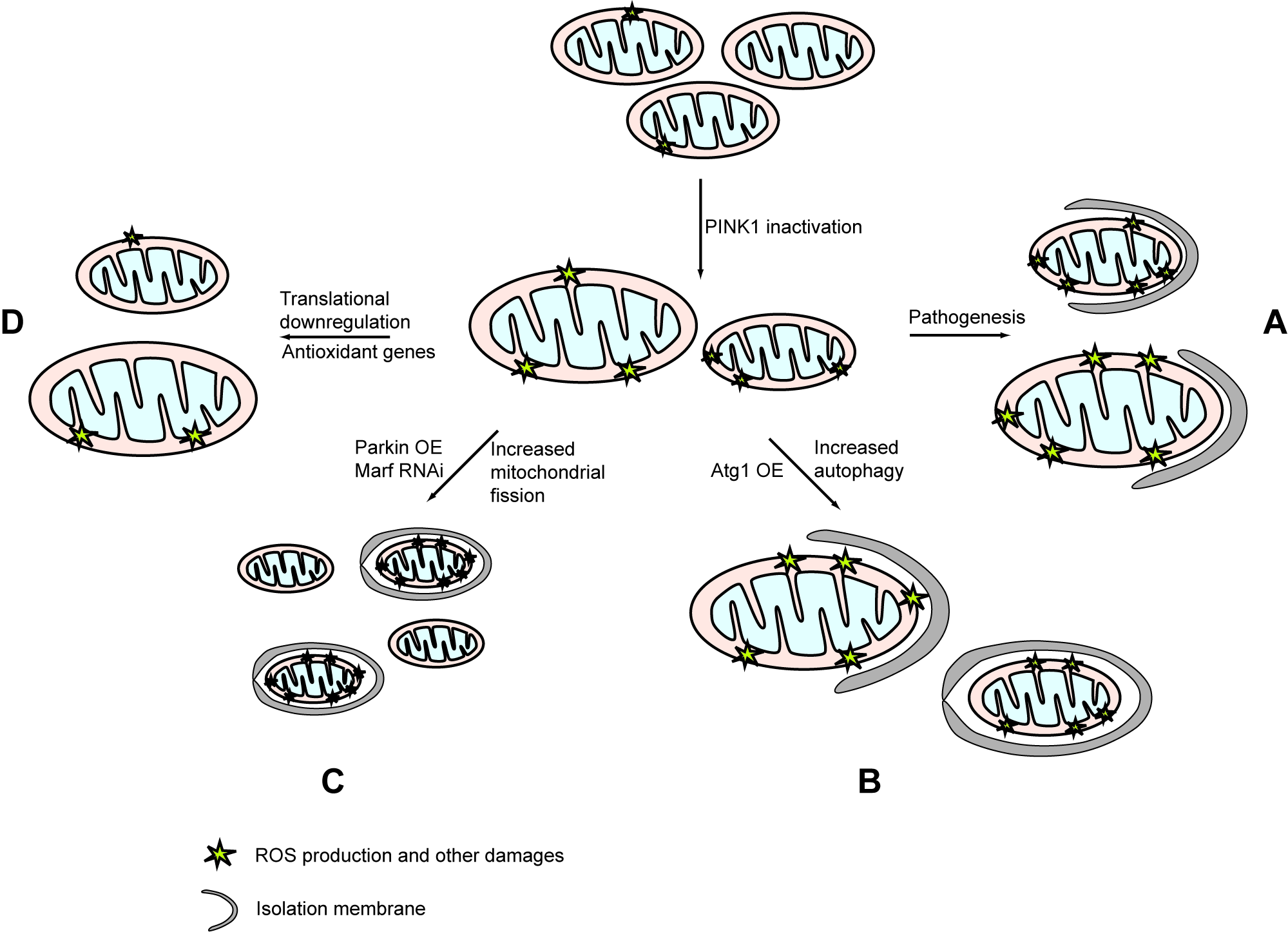

Supplement: Figure S3 — Pathways that can affect PINK1 pathogenesis. PINK1 inactivation generally leads to the accumulation of dysfunctional, enlarged mitochondria. (A) Without intervention, those dysfunctional mitochondria continue to generate reactive oxygen species (ROS) and accumulate damages that lead to mitochondrial death, energy depletion and tissue degeneration. Although autophagy is induced as a compensatory response to PINK1 inactivation, this native response may not be strong enough to allow isolation membrane to efficiently engulf the entire damaged mitochondrion. (B) When autophagy is further induced mildly by Atg1 OE, the generation of more isolation membrane may allow the autophagosomes to form around the entire mitochondrion. However, this process may still be inefficient, with some large dysfunctional mitochondria left unremoved. (C) The efficiency of mitophagy may be further increased when enhanced mitochondrial fission breaks the enlarged mitochondria into smaller ones to allow easy access, expansion of isolation membrane and the maturation of autophagosome. Mitochondrial fission may also selectively segregate damaged part of mitochondria from the healthy one, increasing the specificity and efficacy of mitophagy. The increase in mitochondrial fission could be achieved by direct manipulation of mitochondrial fusion/fission machinery, such as knocking down Marf, or by the overexpression of Parkin, which has been shown to reduce Marf protein level through ubiquitination. However, Parkin OE and Marf RNAi likely exert more functions than simply facilitating autophagy in PINK1 mutant background, since blocking autophagy does not completely eliminate their protective effects. (D) The dysfunctional mitochondrial phenotype due to PINK1 inactivation can also be ameliorated by increasing the expression of antioxidant genes or decreasing protein translation. Antioxidant genes rescue PINK1 mutant phenotype by direct scavenging of ROS, while decreased protein translation acts through [file pgen.1001237.s003.tif]
